# Valorization of Coffee Cherry By-Products Through Fermentation by Human Intestinal Lactobacilli in Functional Fermented Milk Beverages

**DOI:** 10.3390/foods14010044

**Published:** 2024-12-27

**Authors:** Antonia Picon, Yolanda Campanero, Carmen Sánchez, Inmaculada Álvarez, Eva Rodríguez-Mínguez

**Affiliations:** 1 Departamento de Tecnología de Alimentos, INIA, CSIC, Carretera de La Coruña Km 7, 28040 Madrid, Spain; 2 Unidad de Servicio de Técnicas Analíticas, ICTAN, CSIC, Calle José Antonio Novais 6, 28040 Madrid, Spain

**Keywords:** coffee cherry pulp, lactobacilli, antioxidant capacity, antimicrobial activity, fermented milk, starters, fermented foods, functional foods

## Abstract

During coffee production, the removal and disposal of the coffee bean-surrounding layers pose an environmental problem. In this work, we examined the effects of several aqueous coffee cherry extracts on the growth and metabolism, biofilm formation, antioxidant capacity and antimicrobial activity of six lactobacilli from the INIA collection and a commercial probiotic *Lactobacillus rhamnosus* GG strain. Growth medium supplementation with different coffee cherry extracts (at 40%) stimulated strain growth and metabolism. The ground cherry pulp extract (CPE) with the highest total polyphenol content was selected for further use. This CPE contained alkaloids, phenolic acids and flavonoids. Upon CPE supplementation, some strains significantly (*p* < 0.01) increased biofilm formation, while all strains increased antioxidant capacity and antimicrobial activity. After preliminary tests, we developed three bifunctional dairy products, containing 20% CPE and fermented with strains INIA P495, INIA P708 or GG. These strains maintained high levels after manufacture, refrigerated storage, and throughout an in vitro procedure mimicking gastrointestinal tract conditions. Compared to controls, CPE-containing products showed increased levels of total polyphenol compounds, antioxidant capacity and antimicrobial activity, together with positive sensory characteristics. CPE and these selected strains could thus be used to elaborate innovative functional fermented milk products.

## 1. Introduction

Coffee is one of the most widely consumed beverages in the world. The estimated total world coffee production in 2022 was 10.8 million tons [1]. The coffee sector is still growing due to increasing consumption in emerging economies, a strong interest in specialty coffee and product innovations in developed countries [2]. The two major coffee species consumed worldwide are *Coffea arabica* (Arabica) and *Coffea canephora* (Robusta). Arabica coffee is considered to be superior to Robusta due to its organoleptic properties [3]. Interest in *C. canefora* has been steadily growing, however, due to its considerable resistance to climate change [4].

Relevant factors determining the coffee quality include agricultural procedures (mostly genetic makeup of the beans and their ripeness, cultivation and harvesting methods, seasonality, altitude and climate), processing methods, bean roasting and grinding, as well as beverage preparation and consumption [5]. Since coffee is produced from roasted beans, the surrounding layers (silver skin, parchment, mucilage, pulp and skin) have to be removed and are discarded as waste [6]. Unsafe disposal of the corresponding waste has a negative impact on the environment in coffee-producing countries, due to high acidity and caffeine, polyphenol and tannin concentrations [7]. To improve the sustainability of coffee production, bioactive compounds such as alkaloids, melanoidins and polyphenols could be obtained from these by-products and used for the development of nutraceuticals, functional foods or cosmetics [6]. Several reviews dealing with different aspect of coffee by-product valorization have been recently published, providing compositional data, their incorporation as ingredients in food formulations and new product development [8], application of fermentation in the coffee industry [9], and as prebiotic ingredients [10]. These reviews highlight the great industrial potential of coffee by-products.

Polyphenol compounds are secondary plant metabolites with several protective roles for humans. They may act locally against oxidative damage at the gastrointestinal tract (GIT), and systemically by reducing the risk of several chronic diseases [11]. Polyphenols are classified as prebiotics, i.e., substrates that are selectively utilized by host microorganisms conferring health benefits by the International Scientific Association for Probiotics and Prebiotics [12]. Therefore, dairy product health benefits could be further improved by supplementation with polyphenol-rich extracts [13]. 

Coffee microbiota mostly comprise bacteria (lactobacilli, *Bacillus*, *Arthrobacter*, *Acinetobacter*, *Klebsiella* and *Weissella*) and yeast (*Saccharomyces*, *Pichia*, *Candida*, *Rhodotorula*, *Hanseniaspora* and *Kluyveromyces*) [14]. During coffee processing, coffee microbiota plays a pivotal role in fermentation, significantly impacting the coffee’s chemical composition and sensory attributes [5].

Moreover, lactobacilli have a prominent role in fermented dairy products [15]. They are recognized as human gut microbiota commensals and have been used as probiotics for a long time [15]. Lactobacilli are able to transform complex dietary polyphenol compounds, increasing their bioavailability [16]. Depending on individual microbiota composition, however, great variations in polyphenol transformation have been observed [17]. 

Consumers with limited endogenous polyphenol-transforming abilities in particular could benefit from the supplementation of dairy products with polyphenol-rich extracts. Fermentation with transformation-capable intestinal lactobacilli strains could thus provide a cost-effective method to reach a wide public.

The aims of this work were: (1) to examine the effects of aqueous coffee cherry extracts on several lactobacilli strains (most of them from intestinal origin), and (2) to develop bifunctional dairy products containing a coffee cherry pulp extract (CPE) and one of the strains able to increase its polyphenol bioavailability.

## 2. Materials and Methods

### 2.1. Coffee Cherry Extract Preparation and Preliminary Characterization

The coffee (*Coffea canephora*) cherries used in this work were collected and sun-dried by local farmers for Café au Light Group S.L. (Madrid, Spain) in Guinea Conakry during the 2022 harvest season. After air transport, washing and drying, they were vacuum packaged and stored at –30 °C until processing.

After thawing, pulp and bean were mechanically separated. Part of the pulp was ground in a coffee grinder (Jata, Model ML132, Tudela (Navarra), Spain) to a homogeneous powder with a particle size of less than 2 mm. Four types of extraction were performed, each with 10 volumes of sterile distilled water (in a similar way to preparing an herbal tea). For the first two, whole pulp or ground pulp were mixed with boiling water and kept at room temperature for 7 min. For the third method, ground pulp was mixed with sterile water at 4 °C and kept at this temperature for 30 min. For the last method, beans were mixed with boiling water and kept at room temperature for 7 min. Extracts were passed through a stainless-steel strainer and filtered through a 0.22 µm Cytiva Whatman GD/X filter (Global Life Science Solutions Operations UK Ltd., Little Chalfont, Buckinghamshire, UK). 

To test extract sterility, in duplicate, 50 µL portions of each extract were spread on plate count agar (PCA, Difco, Beckton, Dickinson & Co, Le Pont de Claix, Francia) and yeast glucose chloramphenicol agar (YGC, Difco, Beckton, Dickinson & Co, Le Pont de Claix, Francia) plates. Incubations were performed at 30 °C for 48 h for PCA plates and at 25 °C for five days for YCG plates.

Extract pH and absorbance at 600 nm (A_600_, as an indication to color differences) were measured, in triplicate, with a Beckman DU 650 spectrophotometer (Beckman Instruments S.A., Madrid, Spain) and a Crison pH-meter (model Basic 20, Crison Instruments, Barcelona, Spain), respectively. Following the method of Velioglu et al. [18], we used gallic acid and the Folin-Ciocalteau reagent (both from Sigma-Aldrich Co., St. Louis, MI, USA.) to determine, in triplicate, the total polyphenol content (TPC). The results are represented as µg of gallic acid equivalents (GAE) per mL.

### 2.2. Strains Used and Growing Conditions

Six strains, part of the INIA culture collection, were used in this work. Five strains, *Lacticaseibacillus (Ls.) paracasei* ssp. *paracasei* INIA P495 and INIA P708, *Ls. rhamnosus* INIA P334, *Lactobacillus gasseri* INIA P508, and *Limosilactobacillus mucosae* INIA P459 originated from breast-fed infants [19]. These five strains survived the major GIT conditions well and showed a broad inhibitory activity and limited coaggregation with bacterial pathogens. Also, these strains were susceptible to beta-lactamase inhibitors and protein synthesis inhibitors while not bearing mucinolytic activity or producing biogenic amines [19]. Strain INIA P459 possessed bile salt hydrolase activity and produced hydrogen peroxide [19]. Strain INIA P708 possessed a ropy phenotype and produced exopolysaccharides (EPS). The sixth strain, *Lactiplantibacillus (Lp.) plantarum* ssp. *plantarum* INIA TAB84, was a dairy isolate that produces an uncharacterized plantaricin [20]. As reference, the commercial probiotic *Ls. rhamnosus* GG strain [21] was included in the study.

Strains were anaerobically (Anaerogen, Oxoid, Basingstoke, UK) grown at 37 °C for 24 h on plates of de Man, Rogosa and Sharpe medium (MRS, Difco) with 0.05% cysteine-HCl (Sigma-Aldrich Co.) (MRSC) and 1.5% agar (Difco). 

### 2.3. Effect of Coffee Cherry Extracts on Lactobacilli Metabolism

To test metabolic activity, we followed the procedure developed in our previous study [22]. Briefly, brain heart infusion (BHI, Oxoid) medium with 0.05% cysteine-HCl, 0.03% methyl blue (Sigma-Aldrich Co.) and 1.5% agar (Difco) was dissolved in 60% of the total water volume and sterilized. One of the extracts, or water (control plates), was added aseptically to make up the total volume. Solidified plates were stored at 4 °C until use. 

Drops (5 µL) of bacterial cell suspensions (in sterile 0.2% peptone water with 0.05% cysteine-HCl, at approximately 8.5 log CFU/mL) were placed on plates containing individual coffee cherry extracts or no extract (control plates), in duplicate. After anaerobic incubation at 37 °C for 24 h, bacterial growth and dye color alteration were recorded, following the code described in [22]: 0 = no growth, 1 = faint growth without color change, 2 = growth and dye change to blue, 3 = growth and a blue ring of up to 2 mm around the drop and 4 = growth and a blue ring of more than 2 mm around the drop.

### 2.4. Preparation of the Coffee Cherry Pulp Extract (CPE)

The coffee cherry pulp was placed in a kitchen robot (Thermomix, model TM31, Vorwerk España M.S.L., S.C., Madrid, Spain) and subjected to blade speeds of 6 for 60 s and 8 for 30 s. Approximately 70 g were weighed in each of the two glass beakers, mixed with 10 volumes of boiling water and incubated for 7 min in a stove at 50 °C (Memmert GmbH, Schwabach, Germany). The mixture was briefly stirred, and particulate material was removed by filtering through a sterile gauze. Microbial sterility was achieved by vacuum filtration through a 0.2 µm polyethersulfone membrane (TPP Techno Plastic Products AG, Trasadingen, Switzerland). Sterile 50 mL amber plastic tubes (Eppendorf AG, Hamburg, Germany) were used to store extract aliquots at –40 °C.

### 2.5. CPE Characterization

Extract sterility, pH, TPC and A_600_ were determined as described in Section 2.1. CPE dry matter was determined as percentage of the initial weight. The total sugar content was measured by the colorimetric phenol-sulfuric acid method [23] using glucose (Sigma-Aldrich Co.) as a standard, and protein content was assayed with a Coomasie Plus (Bradford) reagent kit (Thermo Scientific, Rockford, IL, USA) and bovine serum albumin (BSA) as standard [22]. The Benzie & Strain method [24], with modifications and Trolox (Sigma Aldrich Co.) as standard, was followed to determine the CPE antioxidant capacity [22]. All determinations in this section were performed in triplicate.

In order to identify polyphenol compounds, the CPE was subjected to HPLC-PAD/ HPLC-ESI-MS, at the ICTAN (CSIC) Analysis Service Unit, following the method and equipment previously described [22]. Analyses were carried out in duplicate.

### 2.6. CPE Effect on Lactobacilli Growth and Metabolism

Growth experiments were performed in BHI broth with 0.05% cysteine-HCl (BHIC) to analyze, in a quantitative manner, the effect of the CPE on strain growth and survival. BHIC broth was supplemented with 40% CPE, while broth without CPE served as control. Strains were inoculated in broth at an approximate level of 7.5 log CFU / mL and incubated under anaerobic conditions at 37 °C for 6 days. Microbial levels, pH and total polyphenol content were determined at days 1 and 6.

### 2.7. CPE Effect on Lactobacilli Biofilm Formation

To study the effect of CPE on biofilms, we followed the method of Lee et al. [25], with some modifications [22]. Briefly, 200 µL of BHIC broth containing 0.02% oxgall (Sigma-Aldrich Co.) and CPE or water (control) were placed in each well of 96-multiwell plates (Thermo Fisher Scientific, Roskilde, Denmark) and inoculated with 2 µL of bacterial suspensions (prepared as described in Section 2.3). For each strain, twelve replicates, divided over two independent experiments, were prepared. Non-inoculated wells were included as negative controls. After 24 h of anaerobic incubation at 37 °C, the liquid phase was discarded, and the wells stained with 0.4% crystal violet solution for 30 minutes. After washing thrice with distilled water, biofilms were resuspended in 30% glacial acetic acid and quantified by reading A_600_ in a microplate reader (Multiskan Spectrum, Thermo Fisher Scientific Oy, Vantaa, Finland).

### 2.8. CPE Effect on Lactobacilli Antioxidant Capacities

The antioxidant capacity of the strains was evaluated by measuring the reduction of the 2,2-diphenyl-1-picrylhydracyl (DPPH, Sigma-Aldrich Co.) radical [26]. Strains, grown on BHIC plates, supplemented or not with 40% CPE, were suspended in 0.85% NaCl and adjusted to an OD_600_ of 1.2. In triplicate, cell suspensions (400 µL) were mixed with DPPH 0.2 M (500 µL) and incubated protected from light at room temperature for 30 min. After centrifugation at 6026 g for 3 min, A_517_ was measured. A 0.85% NaCl solution (400 μL) was used as blank. Total antioxidant capacity (TAC) of each culture was calculated as the percentage of DPPH reduction: TAC = [1 − (A_517_ (sample)/A_517_ (blank))] × 100.

### 2.9. CPE Effect on Lactobacilli Antimicrobial Activities

CPE antimicrobial activity toward four pathogenic strains of the INIA collection was studied, in a similar way as previously described [22]. Gram-positive strains *Clostridium perfringens* CECT 486 (C) and *Listeria monocytogenes* Scott A CECT 5672 (L) and Gram-negative strains *Klebsiella oxytoca* INIA col 108 (K) and *Salmonella enterica* serovar Enteritidis CECT 4155 (S) were tested. Strains were grown in BHI at 37 °C for 24 h under anaerobic conditions (*Clostridium perfringens* CECT 486) or as stand cultures.

The stand-alone inhibitory activity of the CPE on pathogenic strains was evaluated following the method previously described [22]. First, 5 mL of 50 °C-tempered 0.5% BHI soft agar, previously inoculated with fresh cultures of each pathogenic strain at a 0.1% (final concentration, approx. 5.5–6 log CFU/mL), were layered on top of solidified BHI plates, in duplicate. Next, 5 mm-diameter wells were bored with a sterile punch. Finally, each well was filled with the serially diluted CPE extract (100%, 50%, 40%, 20%, 10%, 5%, 1%, 0.5%, 0.25%, 0.125% and 0.0625%; 50 µL per well). Plates were incubated at 37 °C for 24 h.

To study the combined antimicrobial activity of the selected strains (four intestinal strains plus the GG reference strain) and CPE against pathogenic strains, suspensions of cells grown on BHIC (control) or BHIC + 40% CPE plates were prepared as described in Section 2.3. Drops (5 µL) of each suspension were deposited on a new BHI agar plate. After anaerobic incubation at 37 °C for 24 h, a layer of a 50 °C-tempered inoculated BHI soft agar, previously inoculated with fresh cultures of each pathogenic strain (prepared as explained above), was poured on top. Plates were examined for the presence of inhibition halos after 24 h incubation at 37 °C.

### 2.10. Manufacture of CPE Fermented Milk Products

Before starting manufacturing, a group (n = 8) of trained panelists performed a preliminary test to choose a extract concentration suitable for sensory evaluation. UHT milk (Pascual, Burgos, Spain) samples supplemented with 20 or 40% CPE extracts were tested. 

For the manufacture, two independent experiments were performed. UHT milk (Pascual, Burgos, Spain) was supplemented with 20% CPE or sterile water (control) and distributed in 15 ml sterile polypropylene tubes (VWR, Radnor, PA, USA). Control and CPE-supplemented milk tubes were inoculated at 1% with each cell suspension (prepared as in Section 2.3) of the two selected strains (*Ls. paracasei* subsp. *paracasei* INIA P495 and INIA P708) and the reference strain, *Ls. rhamnosus* GG, at a final concentration of approximately 7.5 log CFU/mL. Non-inoculated control tubes were included. All tubes were anaerobically incubated at 37 °C for 24 h. Fermented milk products were introduced in a cold chamber (4 °C) and stored for up to 20 days.

### 2.11. CPE Fermented Milk Product Characterization 

Fermented milk products were analyzed after 1, 12 and 20 days. Microbial levels were determined in duplicate, as described in Section 2.2. To test strain survival *in vitro* under major GIT conditions, the method developed by Langa et al. [27] was applied. Briefly, fermented dairy product samples (1 mL) were mixed with a pH 3.0 buffered PBS solution (9 mL) at 37 °C for 90 s. Mixtures were anaerobically incubated at 37 °C for 1 h. Thereafter, mix samples (1 mL) were added to 9 mL of bile solution (0.15%, ox-bile desiccated; Oxoid) and kept anaerobically at 37 °C for 1 h, before microbial enumeration. Product antimicrobial activities were tested as described in Section 2.9.

Fermented dairy product pH values were determined in triplicate. Following the method of del Olmo et al. [28], polyphenol compounds were extracted. Briefly, samples (1 mL) were subjected to a first extraction with an HCl-acidified (16 mM) methanol:water (50:50) solution (9 mL), and a second one with an acetone:water (70:30) solution (9 mL). Both extractions were performed under constant agitation at room temperature for 1 h. Extracts were mixed to a 1:1 proportion and filtered (0.22 µm PVDF membrane filter, Millex-GV, Merck Millipore Ltd., Cork, Ireland). Filtrates were used to determine, in triplicate, fermented dairy product TPC and antioxidant capacity, as described in Section 2.1 and Section 2.5, respectively.

After 24 h of refrigerated storage, a group of trained panelists (n = 8) performed a preliminary sensory test. Panelists received information about the samples, procedures and their rights. We collected their signed written consent to participate. For the descriptive test, a specific sensory lexicon was developed [29], and thereafter, panelists were asked to describe aspect, odor and flavor characteristics.

### 2.12. Statistical Analysis

Two-way analysis of variance (ANOVA) with presence/absence of CPE and strain as main effects (SPSS 25.0 statistical package, IBM Corporation, Armonk, NY, USA) was applied to data. Tukey’s test by one-way ANOVA, with significance assigned at *p* < 0.01, was then performed to compare means (presence/absence of CPE or strain).

## 3. Results and Discussion

### 3.1. Coffee Cherry Extracts: Preliminary Characterization and Selection

Four coffee cherry extracts, three from pulp and one from bean, were obtained and evaluated (Table 1). Pulp extracts displayed significantly (*p* < 0.01) lower pH, A_600_ and TPC values than bean extract. Among the extracts, the ground pulp prepared with boiling water showed the highest TPC. 

The results on the effects of coffee cherry extracts on strain metabolism are shown in Table 2. Bacterial growth was qualitatively recorded in all plates after 24 h incubation. All strains showed limited growth and dye color change in control plates and in plates with the bean extract. 

However, the three pulp extracts stimulated strain metabolism, showing blue rings (due to acid production) of bigger diameters than in the control plates. The best scores were recorded for strains grown with ground pulp extracts, irrespective of extraction temperature. The biggest stimulations were recorded for strains INIA P495, INIA P708 and the GG reference strain. 

In contrast to what we observed in this work, with 40% concentrations well tolerated by all lactobacilli strains, extract concentrations of mangosteen fruit above 10% were detrimental for the growth of these strains [22].

Taking into account all these results, the ground pulp extract prepared with boiling water was selected and prepared at a large scale.

### 3.2. CPE Characterization

The physico-chemical characteristics for this coffee cherry pulp extract are shown in Table 3. Both pH and A_600_ values were similar to the ones obtained for the ground pulp extract prepared with boiling water. Sucrose is the most abundant sugar present in coffee; it has an important contribution to the taste of this beverage [30]. A raw filtrate of the wet coffee reached a sugar content of 1.67 mg GE/mL [31]. Taking into account that the ratios used in the wet processing were 1 Kg pulp:80–120 L water [31], and that we used a 1:10 ratio, the total sugar content of our CPE was lower than expected. These differences could be explained, however, by the large variations in sucrose found between diverse species and origins and even within beans from the same batch [30]. In comparison with previous data on mangosteen extracts [22], CPE had a lower sugar content than mangosteen pulp extract (12.14 mg GE/mL) or even mangosteen rind extract (9.91 mg GE/ml). 

The higher TPC of the CPE, in comparison to the ground pulp extract previously prepared, might be due to the higher extraction temperature of 50 °C vs room temperature [32]. The TPC of the CPE was lower than the 1.3 mg GAE / mL obtained for the raw filtrate of wet coffee processing [31]. However, TPC on a weight basis (4.06 mg GAE/g fresh pulp) was in agreement with the 4.9 mg GAE/g pulp DM [33]. Compared to the mangosteen extracts of our previous work [22], CPE contained a much lower TPC (5.70 and 10.01 mg GAE / mL for pulp and rind, respectively). Accordingly, the CPE antioxidant capacity was lower than those of the mangosteen extracts (100.95 and 186.60 mM TE for pulp and rind, respectively). 

**Figure 1:**
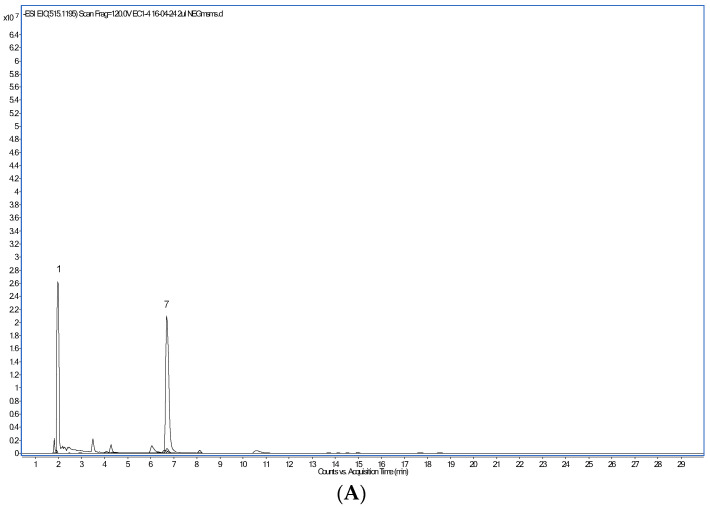

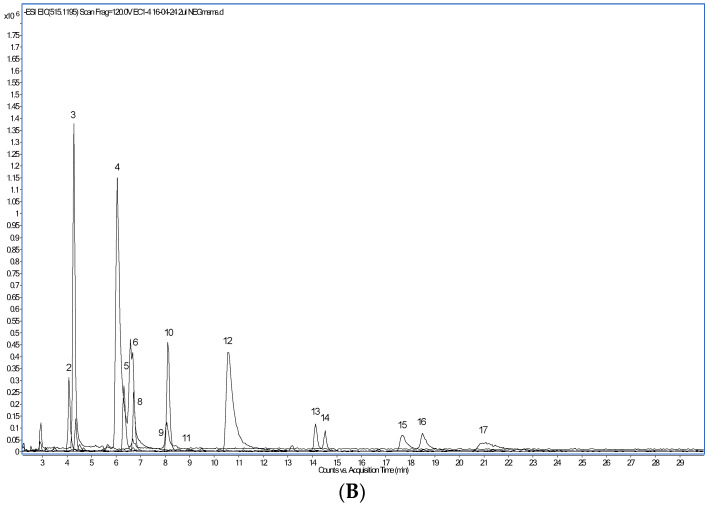
Extracted ion chromatogram of major polyphenol compounds detected in the coffee cherry pulp extract. (**A**): compounds detected after operation in the positive ion mode. (**B**): compounds detected after operation in the negative ion mode.

The most abundant phenolic compounds detected in the CPE by HPLC-PAD/HPLC-ESI-MS (Figure 1) were, in decreasing amounts (Table 4), the alkaloids trigonelline and caffeine, protocatechuic and chlorogenic acids, caffeoylquinic and feruloylquinic acids, the flavan-3-ol catechin and, in lower quantities, caffeic acid, the flavonoid glycoside rutin, several dicaffeoylquinic acid isomers and epicatechin. Following the work of Clifford et al. [34], the isomeric form of each caffeoylquinic, feruloylquinic and dicaffeoylquinic acids was assigned. Trigonelline and caffeine have been previously identified in aqueous arabica and robusta coffee extracts [35,36]. A higher level of trigonelline as compared to caffeine was found, in agreement with published results [37]. Trigonelline biosynthesis occurs in the pericarp of coffee berries, whereas caffeine is predominantly produced in the beans [37]. Interestingly, the NAD^+^ precursor trigonelline seems to improve muscle function during ageing [38]. Comparable to our findings, protocatechuic and chlorogenic acids were the dominant phenolic compounds identified in a beverage prepared with dried coffee cherry pulp and hot water [33]. Several caffeoylquinic, feruloylquinic, and dicaffeoylquinic acid isomers have been found by others, too [3,34,36]. However, caffeoylferuloylquinic or p-coumaroylquinic acids [3,33] were not detected in this work.

Flavonoids such as catechin, epicatechin and rutin are found in many plants. These three compounds were also detected in mangosteen extracts [22]. In agreement with our results, all three were reported in an 80% methanol fresh coffee pulp extract [39]. However, only rutin was detected in coffee cherry pulp samples [30]. According to Aravind et al. [11], these compounds have been shown to improve the function of endothelial tissues, alter glucose metabolism, reduce oxidative stress and ameliorate insulin resistance. 

### 3.3. CPE Effect on Bacterial Growth and Metabolism

After 24 h, all tested lactobacilli strains grew just as well in control as in CPE-supplemented BHIC media (Table S1). Strains INIA P495, P708 and P334 obtained the highest values, with levels close to 9 log CFU/mL. Although a stimulatory effect of polyphenols has been described for lactobacilli [40], no significant differences between both growth media, either at day 1 or 6, were recorded. However, pH decreases were significantly bigger (*p* < 0.01) in CPE-supplemented BHIC than in BHIC controls. The highest decreases were recorded for strains INIA P495, INIA P708 and GG, with values around 4.3–4.5, in agreement with observations of solid media (Table 2). Although pH values of 4.5–5.0 typically result in a significant to complete inhibition of growth [41], CPE seemed to protect cultures. We found no differences in growth between CPE-supplemented or control media. Similarly, Pereira et al. [42] showed that inoculation with a *Lp. plantarum* strain resulted in faster and improved coffee bean fermentation, converting pulp sugars into organic acids and reaching pH values of 4.5 in 12 h.

CPE addition significantly (*p* < 0.01) increased TPC, by about 100 μg GAE / mL. However, no increase in TPC values were recorded for any of the tested cultures. While lactobacilli are able to transform complex dietary polyphenols, increasing the bioavailability of these compounds [16,43], the spectroscopic technique used to determine TPC did not seem to be sensitive enough to detect the differences observed by HPLC-ESI/MS [44]. 

### 3.4. Functional and Probiotic Capacities of Selected Strains in the Presence of CPE

For the next experiments, three of the six lactobacilli strains, each showing different survival and metabolic behavior, were selected (INIA P495, INIA P708 and INIA P459). For these isolates, biofilm formation, antioxidant capacity and antimicrobial activity were compared against the commercial probiotic GG strain.

#### 3.4.1. CPE Effect on Lactobacilli Biofilm Formation

Bacterial biofilm formation is increasingly recognized as a passive virulence factor facilitating many infectious disease processes [45]. Microbial communities are embedded in a self-produced matrix of extracellular polymeric substances that mediate attachment to surfaces [46]. For probiotic bacteria, biofilm formation, however, is considered a beneficial trait, as it promotes permanence in the host mucosa and avoids colonization by pathogenic bacteria [46]. The GG strain, with a high biofilm formation capacity, served as positive control to develop the biofilm staining method [25] used in this work. As shown in Table 5, biofilm formation was strain-dependent. In BHIC with 0.02% oxgall (BHICO), three out of the four strains generated biofilms. 

Strain INIA P495 produced significantly (*p* < 0.01) more biofilm matter than the GG strain. Strain INIA P708 did not produce biofilm in the assayed conditions, in a similar way to what we observed for this strain in the presence of mangosteen extracts [22]. In the presence of CPE, the three biofilm-forming strains increased biofilm production. However, this increase was significant (*p* < 0.01) only for strains INIA P459 and GG. These data are relevant, because they suggest that nutritional habits influence intestinal permanence of a strain. Opposite to what we observed, others reported anti-biofilm activities of cranberry polyphenols against oral streptococci [47] and of caffeine against pathogenic *E. coli* [48]. Also, mangosteen extracts seemed to produce an anti-biofilm effect in strains INIA P459, INIA P495 and GG [22].

#### 3.4.2. CPE Effect on Strain Antioxidant Capacity

After 24 h, an increase of around 3-fold in antioxidant capacity with respect to control conditions was recorded for all strains grown in the presence of CPE (Table 6). The highest increase (more than 4-fold) was recorded for the GG strain. After 6 days, a significant (*p* < 0.01) increase in antioxidant capacity with respect to day 1 was recorded for all strains except for strain GG. The latter maintained an antioxidant capacity level similar to the one recorded at day 1. As expected, higher DPPH reduction levels (close to 90%) were recorded for all four strains when grown in the presence of mangosteen extracts [22]. 

Polyphenol compounds have a protective role against oxidative damage and act as hydrogen donors and metal ion chelators [11]. The higher antioxidant capacities found with CPE could be attributed to the action of lactobacilli enzymes, mainly glycosidases and esterases, liberating aglycones and free phenolic acids. These transformed compounds have higher antioxidant activities than the parental compounds [43]. The increased antioxidant activity observed for the investigated intestinal bacteria indicated a potential effect of coffee cherry pulp extract on the lactobacilli of our intestinal microbiota. Moreover, by limiting the excessive amounts of reactive radicals *in vivo*, these strains may play a role in the prevention and control of oxidative stress-associated diseases [49].

#### 3.4.3. CPE Effect on Intestinal Lactobacilli Antimicrobial Activity Against Pathogenic Strains 

To assay the effect on pathogenic bacteria, overlays of four pathogenic strains on plates precultured with intestinal lactobacilli were used. None of the assayed pathogenic strains was inhibited when growing in BHIC + 40% CPE. Moreover, the studied strains had a limited antimicrobial activity against *Listeria monocytogenes* (Table 7). However, in combination with the CPE, the inhibition against *L. monocytogenes* was greatly improved. Also, an increased inhibitory effect against the other three pathogenic strains was observed. Strain INIA P708 in combination with CPE inhibited all assayed pathogens with the biggest inhibition zones. In other studies, a CPE that contained chlorogenic acid and caffeine as the most prominent compounds presented antibacterial activity against both Gram-positive (*Staphylococcus aureus* and *S. epidermidis*) and Gram-negative bacteria (*Pseudomonas aeruginosa* and *Escherichia coli*) [35]. The disruption of cell membranes by phenolic acids alters numerous intracellular functions and effectively causes cell death [35]. In contrast, we observed in a previous study that mangosteen extracts resulted in limited antimicrobial activity and only against Gram-positive pathogenic strains [22].

Diets rich in fermented food, typically those that contribute to intestinal lactobacilli, were shown to increase microbiota diversity and decrease inflammatory markers over time [50]. Recently, *in vivo* studies suggested that coffee has modulating impacts on gut microbiota. Coffee seems to increase the relative abundance of beneficial bacterial phyla such as Firmicutes and Actinobacteria while decreasing Bacteroidetes [51]. Finally, coffee also increases *Bifidobacterium* spp. abundance but suppressed Enterobacteria [51].

### 3.5. Manufacture and Characterization of CPE Fermented Milk Products 

The sensory characteristics of whole UHT milk supplemented with two CPE concentrations (20 or 40%) were determined before starting manufacture. The flavor of milk samples with 20% CPE was described as milky, slightly herbaceous and aromatic. In contrast, the flavor of the sample with 40% CPE was described as strongly herbaceous, sweeter and more astringent and persistent than that of milk with 20% CPE. Since the flavor of milk with 20% CPE was considered equilibrated and not causing dryness, we selected this concentration for the manufacture of fermented milk products.

From here on, whole UHT milk, plain or with 20% CPE, was aliquoted and fermented with selected strains. Since only strains INIA P495, INIA P708 and GG conferred adequate sensory characteristics, these three strains were chosen for the manufacture of the fermented milk products. Table 8 shows microbial levels in the fermented products after manufacture and cold storage and after an *in vitro* process mimicking the major GIT conditions. Strains were inoculated at approximately 7.5 log CFU/mL. After fermentation, all strains reached levels higher than 8.0 log CFU/mL. Microbial levels were not significantly different between milk or CPE-supplemented milk. Products with strain INIA P708 reached the highest levels, with values above 9 log CFU/mL. Similar results were obtained before, when strains INIA P708 and GG were used to ferment milk products with or without mangosteen extracts [22] Behavior during storage was strain-dependent: INIA P708 lowered its level in plain milk but maintained it in CPE-supplemented milk. INIA P495 levels increased in plain milk and in milk with CPE after 20 days. Strain GG showed steady levels throughout the whole period in milk as well as in CPE milk fermented products. After 20 days, fermented products with the two INIA strains showed higher levels than products with the GG strain. All fermented milk products reached levels above 7 log CFU/mL throughout manufacture and storage, thereby exceeding the required level (6 log CFU/mL) to offer probiotic health benefits [52]. However, in a fermented coffee brew beverage, the GG strain suffered viability losses, falling below 6 log CFU/mL within 10 weeks at 4 °C, and 3 weeks at 25 °C [53]. While survival to harsh conditions cannot be inferred from culture viability [54], functional and technological strain properties could vary in the presence of different food ingredients [55]. When fermented milk products were exposed to an *in vitro* procedure mimicking major GIT conditions, all three strains survived well in both fermented milk products (Table 8). Similarly, intestinal lactobacilli in the form of pure cultures showed high survival rates to GIT conditions [19], as well as in milk fermented products with mangosteen extracts [22]. Finally, fermented milk products showed similar antimicrobial activities against pathogenic strains as the ones recorded in Table 7.

After fermentation, all strains lowered milk pH (Table 9). As expected, pH decreased significantly (*p* < 0.01) more in milk with CPE than in plain milk. Similar observations were also recorded for fermented milk products with mangosteen extracts and strains INIA P708 and GG [22]. During storage, pH values dropped further in fermented products with the INIA strains, whereas a significant (*p* < 0.01) basification was recorded for the GG strain. This effect was not observed in fermented milk products with mangosteen extracts and GG, probably due to the shorter (15 vs. 20 days) product storage [22]. It is well documented that the GG strain does not grow well in milk due to inability to hydrolyze lactose and casein [56]. The increased lowering of pH observed in milk with CPE was probably due the utilization of sucrose and other sugars present in CPE. 

Milk phenolic compounds are mostly derived from animal feed [28]. Accordingly, we observed that CPE supplementation significantly (*p* < 0.01) increased (by 1.2- to 1.8-fold) the TPC of fermented dairy products (Table 9). These increases, however, were smaller than the 2- or 3-folds recorded for fermented milk products containing 5% mangosteen pulp or rind extracts, respectively [19]. Although no big differences were recorded between days 1 and 12, most of the fermented products obtained significantly (*p* < 0.01) higher TPC levels after 20 days. During storage, strain INIA P708 increased TPC levels 1.3-fold, both in plain milk and milk with CPE. For strain INIA P495, 1.1- and 1.2-fold increases were recorded in plain milk and in milk with CPE, respectively. For the GG strain, no increase and a 1.2-fold increase in TPC levels were recorded in plain milk and milk with CPE, respectively. The increases observed during storage could be due to the action of lactobacilli polyphenol-metabolizing enzymes, leading to the production of new compounds with new biological properties [16,43].

After manufacture, CPE also increased the antioxidant capacity of fermented dairy products around 2.6-fold. Again, this increase was lower than those obtained for fermented milk products with mangosteen extracts (5- and 8-fold for products with mangosteen pulp and rind extracts, respectively) [22]. After 20 days, the antioxidant capacity of plain milk fermented with strains P495 or GG increased in a similar way to that in non-inoculated milk (1.1–1.2-fold), but this increase was bigger for plain milk fermented with strain INIA P708 (1.5 fold). 

The supplementation of products with CPE at the end of storage did not increase their antioxidant capacity (non-inoculated or fermented with strain GG) or increased it slightly (fermented with strains INIA P495 and INIA P708). It is well stablished that several EPS produced by lactobacilli possess antioxidant properties [57]. Therefore, the EPS produced by strain INIA P708 might contribute to the higher values observed for those fermented products. 

As discussed in Section 3.3, although HPLC-ESI/MS [44] detected the transformations that enhance the bioavailability of dietary polyphenols [16,43], spectroscopic techniques were probably unable to reveal the full range of those transformations. Consequently, the CPE fermented milk products may contain bioavailable polyphenols at higher levels than the control or CPE non-fermented milk products. Similar to what we observed using spectroscopic techniques, probiotic fermentation with the GG strain of a coffee brew beverage did not alter TPC or antioxidant capacity [52].

Nowadays, regardless of consumers’ awareness of the relationship between diet and health, food acceptance is still driven by flavor. Panelists found that all CPE fermented milk products were characterized by pleasant odors and flavors (Table 10). In fermented milk products with mangosteen extracts, strains INIA P708 and GG were among those conferring the best sensory characteristics [22].

## 4. Conclusions

The CPE developed in this study positively influenced the growth and metabolism of several lactobacilli strains, including the commercial probiotic GG strain, and improved their antioxidant and antimicrobial properties. Strain levels in CPE fermented milk products increased after manufacture. Strains remained viable during cold storage and after an in vitro process mimicking major GIT conditions. We can conclude that the developed fermented milk products containing CPE and potentially probiotic lactobacilli could have beneficial effects on human health. Therefore, CPE is a suitable candidate to develop novel functional fermented milk products enriched in polyphenol compounds with benefits for human health. Through lactobacilli transformations, the benefits of these polyphenols could be extended to all consumers. The observations in this study may need to be confirmed by performing *in vivo* studies in animal models, and at a further stage, human clinical trials. 

## Figures and Tables

**Table 1:** Preliminary characterization of the coffee cherry extracts ^1^.

Variable	Extracts			
	Whole Pulp	Ground Pulp	Ground Pulp 4 °C	Bean + Silverskin
pH	3.78 ± 0.02 ^b^	3.76 ± 0.01 ^b^	3.74 ± 0.01 ^b^	5.41 ± 0.01 ^a^
A_600_	0.0268 ± 0.0100 ^bc^	0.0460 ± 0.0093 ^b^	0.0193 ± 0.0102 ^c^	0.1352 ± 0.0098 ^a^
TPC ^2^	136.15 ± 1.16 ^c^	314.18 ± 0.61 ^a^	160.48 ± 0.45 ^b^	70.36 ± 0.42 ^d^

^1^ Coffee cherry extracts were prepared with sterile distilled water (at a 1:10 ratio) at 95 °C for 7 min, or at 4 °C for 30 min. ^2^ TPC = Total polyphenol content, in µg GAE/mL. All variables were mean ± SD of triplicate determinations in two experiments (n = 6). Means with lower-case superscripts differ significantly at *p* < 0.01.

**Table 2:** Behaviour of intestinal strains grown on BHI agar with methyl blue (control, BHIC) and supplemented with 40% of the coffee cherry extracts incubated at 37 °C for 48 h under anaerobic conditions.

Strain	Control	Extract			
		Whole Pulp	Ground Pulp	Ground Pulp 4 °C	Bean + Silver Skin
INIA P495	1+	3	4	4	2
INIA P708	1+	3	4	4	2
INIA P334	1	2	3	3	1+
INIA TAB84	1	2	3	3	1
INIA P508	1	2	3	3	1
INIA P459	1	3	3	3	1
GG	1+	3	4	4	1+

Codes: 0 = no growth; 1 = light growth without color change; 2 = growth and change to blue color; 3 = blue ring of up to 2 mm around the drop; 4 = blue ring of more than 2 mm around the drop.

**Table 3:** Characteristics of the coffee cherry pulp extract.

pH	A_600_	DM ^1^ (%)	Sugar Content (mg GE/mL)	Protein Content (μg BSA/mL)	TPC ^2^ (μg GAE/mL)	Antioxidant Capacity (mM TE)
3.81 ± 0.01	0.0451 ± 0.01	1.98 ± 0.03	8.28 ± 0.21	47.39 ± 4.37	406.43 ± 1.50	8.36 ± 0.09

^1^ DM = dry matter and ^2^ TPC = Total polyphenol content. All variables were mean ± SD of triplicate determinations in two experiments (n = 6).

**Table 4:** LC-MS/MS characteristics of major polyphenols detected in the coffee cherry pulp extract.

Peak Number	Retention Time (min)	Compound	[M-H]- m/z	MS2 Ions m/z
1	1.9	Trigonelline	138.0550 *	138 (100), 94 (63), 53 (27), 78 (18), 65 (13)
2	4.1	3-Caffeoylquinic acid	353.0878	191 (100), 135 (35)
3	4.4	Protocatechuic acid	153.0193	109 (100)
4	6.1	Chlorogenic acid (5-Caffeoylquinic acid)	353.0878	191 (100)
5	6.3	Catechin	289.0718	203 (100), 245 (87), 109 (65), 221 (64), 164 (57), 123 (45), 227 (38), 97 (37), 150 (36), 80 (32), 186 (32), 138 (31), 211 (25), 175 (23)
6	6.6	4-Caffeoylquinic acid	353.0878	191 (100), 173 (100), 135 (34)
7	6.7	Caffeine (1,3,7-Trimethylxanthine)	195.0877 *	138 (100), 195 (41), 110 (19)
8	6.7	3-Feruloylquinic acid	367.1035	193 (100), 134 (19)
9	8.1	Caffeic acid	179.0350	135 (100), 105 (12), 93 (12)
10	8.1	5-Feruloylquinic acid	367.1035	367 (100)
11	8.9	Epicatechin	289.0718	203 (100), 245 (87), 109 (65), 221 (64), 123 (45), 227 (38), 97 (37), 150 (35), 186 (32), 80 (32), 289 (31), 138 (31), 211 (25)
12	10.5	4-Feruloylquinic acid	367.1035	191 (100), 173 (24)
13	14.1	Rutin (Quercetin-3-O-rutinoside)	609.1461	609 (100), 300 (11)
14	14.5		609.1461	609 (100), 300 (17)
15	17.7	3,4-di-O-caffeoylquinic acid	515.1195	173 (100), 353 (83), 515 (37), 335 (19), 155 (13)
16	18.5	3,5-di-O-caffeoylquinic acid	515.1195	353 (100), 191 (71), 179 (14), 509 (10)
17	21.1	4,5-di-O-caffeoylquinic acid	515.1195	353 (100), 173 (69), 311 (14), 516 (10), 263 (10)

* Major polyphenol compounds detected in the positive ion mode [M+H]^+^.

**Table 5:** Biofilm formation by the studied strains in medium BHIC with 0.02% oxgall (BHICO) and BHICO supplemented with 40% of the coffee cherry pulp extract.

Strain	A_600_	
	BHICO	BHICO + 40% CPE
INIA P495	0.77 ± 0.12 ^a A^ 0.03 ± 0.01 ^a D^ 0.13 ± 0.02 ^b C^ 0.39 ± 0.09 ^b B^	0.97 ± 0.17 ^a B^ 0.04 ± 0.02 ^a C^ 0.19 ± 0.04 ^a C^ 1.26 ± 0.29 ^a A^
INIA P708		
INIA P459		
GG		

Values expressed as means ± SD of sextuplicate determinations in two experiments (n = 12). Means with different superscripts (lower-case ones within the same row and upper-case ones within the same column, in the same group) differ significantly at *p* < 0.01.

**Table 6:** Antioxidant activities of the studied strains grown with 40% of the coffee cherry pulp extract.

Strain	Time	Percentage of DPPH Reduction	
		Control BHIC	BHIC +40% CPE
INIA P495	1 d	14.70 ± 1.02 ^b A^	45.90 ± 1.53 ^a B^
	6 d	16.31 ± 0.65 ^b A^	65.13 ± 0.52 ^a A^
INIA P708	1 d	10.61 ± 1.13 ^b B^	30.67 ± 1.05 ^a B^
	6 d	18.20 ± 0.57 ^b A^	54.38 ± 0.52 ^a A^
INIA P459	1 d	12.45 ± 0.86 ^b A^	39.27 ± 2.64 ^a B^
	6 d	13.92 ± 0.42 ^b A^	62.16 ± 0.18 ^a A^
GG	1 d	12.78 ± 0.67 ^b B^	53.10 ± 3.72 ^a A^
	6 d	16.75 ± 0.67 ^b A^	54.53 ± 0.47 ^a A^

Means ± SD from triplicate determinations in two experiments (n = 6). Means within the same row with different lower-case superscripts differ significantly at *p* < 0.01. Means for the same strain within the same column with different upper-case superscripts differ significantly at *p* < 0.01.

**Table 7:** Antimicrobial activity ^1^ of the studied strains against pathogenic strains ^2^ in control BHIC medium and BHIC supplemented with 40% of the coffee cherry pulp extract.

Strain	Control BHIC				BHIC + 40% CPE			
	C	K	L	S	C	K	L	S
INIA P495	-	-	+	-	-	D	+++	+
INIA P708	-	D	+	-	++	++	+++	++
INIA P459	D	-	-	-	D	-	+	-
GG	-	D	+	-	+	D	+++	+

^1^ Codes: - = no halo, D = diffuse halo; + = halo < 7 mm; ++ = halo 7–10 mm; +++ = halo > 10 mm.^2^ C = *C. perfringens* CECT 486; L = *L. monocytogenes* Scott A CECT 5672; K = *K. oxytoca* INIA Col 108; S = *S. enterica* serovar Enteritidis CECT 4155.

**Table 8:** Microbial levels ^1^ in fermented milk products, after elaboration and after 12- and 20-day refrigerated storage, and after subjecting fermented milk products to major conditions encountered in the gastrointestinal tract (GIT).

Strain	Time	Levels in Fermented Milk		Levels After Major GIT Conditions	
		M100	M80:CPE20	M100	M80:CPE20
INIA P495	1 d	8.18 ± 0.08 ^a C^	8.30 ± 0.24 ^a B^	8.45 ± 0.12 ^a A^	8.45 ± 0.20 ^a A^
	12 d	8.38 ± 0.07 ^a B^	8.21 ± 0.08 ^a B^	7.71 ± 0.09 ^a A^	7.73 ± 0.05 ^a B^
	20 d	8.74 ± 0.06 ^a A^	8.71 ± 0.10 ^a A^	8.51 ± 0.19 ^a A^	8.75 ± 0.38 ^a A^
INIA P708	1 d	9.18 ± 0.01 ^a B^	9.27 ± 0.13 ^a A^	8.75 ± 0.16 ^a B^	8.69 ± 0.14 ^a B^
	12 d	9.42 ± 0.05 ^a A^	9.31 ± 0.24 ^a A^	9.33 ± 0.04 ^a A^	9.29 ± 0.06 ^a A^
	20 d	9.09 ± 0.15 ^a B^	9.23 ± 0.03 ^a A^	9.32 ± 0.00 ^a A^	9.28 ± 0.04 ^a A^
GG	1 d	8.51 ± 0.13 ^a A^	8.57 ± 0.24 ^a A^	8.26 ± 0.32 ^a A^	8.26 ± 0.12 ^a A^
	12 d	8.51 ± 0.16 ^a A^	8.57 ± 0.12 ^a A^	8.08 ± 0.00 ^a A^	7.71 ± 0.07 ^a B^
	20 d	8.29 ± 0.27 ^a A^	8.49 ± 0.04 ^a A^	7.91 ± 0.06 ^a A^	8.19 ± 0.14 ^a A^

Codes: M100 = milk; M80:CPE20 = milk supplemented with 20% of the coffee cherry pulp extract. ^1^ Microbial levels were expressed as log CFU/mL. Means ± SD (n = 4). Means with different superscripts (lower-case ones within the same row and upper-case ones within the same column) differ significantly at *p* < 0.01.

**Table 9:** pH values, total polyphenol content (TPC) and antioxidant capacity in fermented milk products, after elaboration and after 12- and 20-day refrigerated storage.

Variable	Strain	Time	M100	M80:CPE20
pH	Non inoculated	0 h	6.58 ± 0.00 ^a C^	6.31 ± 0.01 ^b C^
		1 d	6.51 ± 0.01 ^a D^	6.21 ± 0.00 ^b D^
		12 d	6.61 ± 0.01 ^a B^	6.38 ± 0.01 ^b B^
		20 d	6.67 ± 0.00 ^a A^	6.42 ± 0.01 ^b A^
	INIA P495	1 d	6.15 ± 0.03 ^a A^	5.26 ± 0.02 ^b A^
		12 d	6.11 ± 0.03 ^a A^	4.89 ± 0.01 ^b B^
		20 d	5.92 ± 0.03 ^a B^	4.46 ± 0.01 ^b C^
	INIA P708	1 d	5.16 ± 0.02 ^a A^	4.34 ± 0.00 ^b A^
		12 d	4.84 ± 0.02 ^a B^	4.01 ± 0.01 ^b B^
		20 d	4.56 ± 0.02 ^a C^	3.89 ± 0.01 ^b C^
	GG	1 d	6.12 ± 0.02 ^a C^	5.64 ± 0.01 ^b B^
		12 d	6.30 ± 0.01 ^a B^	5.81 ± 0.01 ^b A^
		20 d	6.35 ± 0.01 ^a A^	5.84 ± 0.02 ^b A^
TPC	Non inoculated	0 h	155.45 ± 10.29 ^b AB^	213.57 ± 8.64 ^a B^
		1 d	157.97 ± 1.59 ^b A^	210.67 ± 4.82 ^a B^
		12 d	143.13 ± 1.82 ^b BC^	199.54 ± 2.41 ^a B^
		20 d	141.79 ± 5.95 ^b C^	231.79 ± 10.90 ^a A^
	INIA P495	1 d	150.82 ± 2.25 ^b B^	221.37 ± 1.89 ^a B^
		12 d	150.23 ± 2.95 ^b B^	214.11 ± 5.67 ^a B^
		20 d	159.70 ± 7.34 ^b A^	269.27 ± 4.15 ^a A^
	INIA P708	1 d	170.99 ± 3.44 ^b C^	215.21 ± 2.15 ^a B^
		12 d	185.99 ± 3.40 ^b B^	214.97 ± 2.36 ^a B^
		20 d	223.85 ± 9.11 ^b A^	280.07 ± 12.02 ^a A^
	GG	1 d	146.84 ± 1.48 ^b A^	215.77 ± 4.05 ^a B^
		12 d	145.77 ± 2.93 ^b A^	209.64 ± 2.82 ^a B^
		20 d	145.49 ± 8.73 ^b A^	254.50 ± 7.32 ^a A^
Antioxidant	Non inoculated	0 h	0.71 ± 0.02 ^b B^	1.76 ± 0.02 ^a A^
capacity		1 d	0.67 ± 0.03 ^b B^	1.69 ± 0.06 ^a B^
		12 d	0.70 ± 0.02 ^b B^	1.54 ± 0.01 ^a C^
		20 d	0.79 ± 0.01 ^b A^	1.64 ± 0.03 ^a B^
	INIA P495	1 d	0.64 ± 0.02 ^b C^	1.67 ± 0.04 ^a AB^
		12 d	0.72 ± 0.01 ^b B^	1.62 ± 0.04 ^a B^
		20 d	0.75 ± 0.01 ^b A^	1.69 ± 0.02 ^a A^
	INIA P708	1 d	0.67 ± 0.02 ^b C^	1.72 ± 0.04 ^a B^
		12 d	0.82 ± 0.02 ^b B^	1.65 ± 0.01 ^a B^
		20 d	0.97 ± 0.04 ^b A^	1.81 ± 0.04 ^a A^
	GG	1 d	0.65 ± 0.03 ^b B^	1.68 ± 0.01 ^a A^
		12 d	0.68 ± 0.00 ^b AB^	1.60 ± 0.02 ^a A^
		20 d	0.72 ± 0.02 ^b A^	1.65± 0.06 ^a A^

Codes: M100 = milk; M80:CPE20 = milk supplemented with 20% of the coffee cherry pulp extract. Values expressed as means ± SD (n = 6). TPC, expressed in µg gallic acid equivalents per mL, and antioxidant activity, expressed in millimolar of Trolox equivalents. Means with different superscripts (lower-case ones within the same row and upper-case ones within the same column) differ significantly at *p* < 0.01.

**Table 10:** Sensory characteristics of fermented milk products supplemented with 20% of the coffee cherry pulp extract and fermented with specific strains.

Product	Aspect	Odour	Flavour
M100	white, liquid	lactic	lactic
M80:20CPE	colour slightly darker than milk, liquid	slightly herbal	aromatic, slightly sweet and astringent, CPE taste
M80:20CPE + INIA P495	colour similar to milk, liquid	yogurt, sweet, toasted, caramel	sweet and toasted. Less astringent and acid, and more herbal than M100:20CPE.
M80:20CPE + INIA P708	colour slightly darker than milk, coagulated, viscous, creamy	yogurt, not herbal	acid, lactic, final astringency
M80:20CPE + GG	colour similar to milk, liquid	mild yogurt	similar to M100:20CPE

Codes: M100 = milk; M80:CPE20 = milk supplemented with 20% of the coffee cherry pulp extract.

## Data Availability

The raw data supporting the conclusions of this article will be made available by the authors on request.

## Supplementary Materials

The following supporting information can be downloaded at: https://www.mdpi.com/article/10.3390/foods14010044/s1, Table S1: Microbial levels, pH and total polyphenol content (TPC, expressed in μg GAE /mL) in cultures of BHI broth (control, BHIC) and BHIC supplemented with 40% of the coffee cherry pulp extract (CPE), inoculated with the selected strains and grown at 37 °C for 6 d under anaerobic conditions.
