# Supplementary material for: Valorization of Coffee Cherry By-Products Through Fermentation by Human Intestinal Lactobacilli in Functional Fermented Milk Beverages
Source: Foods. 2024 Dec 27;14(1):44. doi: 10.3390/foods14010044 (PMC11720464; doi:10.3390/foods14010044)
Supplement: Supplementary file 1 [file foods-14-00044-s001.zip › foods-3370485-supplementary.pdf]

Table S1. Microbial levels, pH and total polyphenol content (TPC, expressed in µg GAE /mL) in cultures of BHI broth (control, BHIC) and BHIC supplemented with 40% of the coffee cherry pulp extract (CPE), inoculated with the selected strains and grown at 37 ºC for 6 days under anaerobic conditions.

Variable	Strain	Time	BHIC	BHIC + 40% CPE
Microbial level	INIA P495	0 h	7.52 ± 0.01 a B	7.52 ± 0.01 a B
		1 d	8.92 ± 0.22 a A	8.86 ± 0.10 a A
		6 d	6.98 ± 0.09 a C	6.76 ± 0.05 a C
	INIA P708	0 h	7.61 ± 0.00 a B	7.61 ± 0.00 a C
		1 d	9.08 ± 0.05 a A	9.18 ± 0.03 a A
		6 d	6.99 ± 0.08 a C	7.72 ± 0.03 a B
	INIA P334	0 h	7.58 ± 0.06 a C	7.58 ± 0.06 a B
		1 d	8.96 ± 0.14 a A	8.89 ± 0.02 a A
		6 d	8.30 ± 0.27 a B	6.61 ± 0.17 a C
	INIA TAB84	0 h	7.35 ± 0.04 a C	7.35 ± 0.04 a B
		1 d	8.47 ± 0.08 a A	8.53 ± 0.10 a A
		6 d	7.55 ± 0.08 a B	7.44 ± 0.09 a B
	INIA P508	0 h	7.71 ± 0.07 a B	7.71 ± 0.07 a B
		1 d	8.27 ± 0.06 a A	8.52 ± 0.13 a A
		6 d	8.12± 0.04 a A	7.96 ± 0.14 a B
	INIA P459	0 h	6.84 ± 0.04 a C	6.84 ± 0.04 a C
		1 d	8.35 ± 0.06 a A	8.35 ± 0.11 a A
		6 d	7.87 ± 0.03 a B	7.87 ± 0.03 a B
	GG	0 h	7.86 ± 0.03 a b	7.86 ± 0.03 a C
		1 d	8.69 ± 0.08 a A	8.84 ± 0.04 a A
		6 d	7.11 ± 0.06 a C	8.10 ± 0.02 a B
pH	Non inoculated	0 h*	7.15 ± 0.01 a A	6.55 ± 0.00 b A
		1 d	6.76 ± 0.01 a C	6.37 ± 0.01 b B
		6 d	6.86 ± 0.02 a B	6.41 ± 0.06 b B
	INIA P495	1 d	4.95 ± 0.01 a C	4.37 ± 0.01 b C
		6 d	5.03 ± 0.02 a B	4.39 ± 0.01 b B
	INIA P708	1 d	4.99 ± 0.01 a B	4.34 ± 0.00 b C
		6 d	5.03 ± 0.03 a B	4.41 ± 0.01 b B
	INIA P334	1 d	5.52 ± 0.02 a C	4.84± 0.03 b C
		6 d	5.64 ± 0.04 a B	4.92 ± 0.03 b B
	INIA TAB84	1 d	5.56 ± 0.02a C	4.97 ± 0.01 b B
		6 d	5.78 ± 0.02 a B	5.00 ± 0.02 b B
	INIA P508	1 d	6.19 ± 0.08 a C	5.16 ± 0.00 b B
		6 d	6.32 ± 0.02 a B	5.06 ± 0.10 b B
	INIA P459	1 d	6.26 ± 0.02 a C	5.13 ± 0.02 b C
		6 d	6.36 ± 0.01 a B	5.18 ± 0.02 b B
	GG	1 d	5.12 ± 0.03 a B	4.47 ± 0.01 b C
		6 d	5.16 ± 0.04 a B	4.50 ± 0.01 b B
TPC	Non inoculated	0 h*	723.53 ± 0.49 b A	854.09 ± 2.67 a AB
		1 d	713.63 ± 30.28 b A	837.99 ± 11.95 a B
		6 d	716.06 ± 5.08 b A	862.19 ± 5.03 a A
	INIA P495	1 d	708.17 ± 8.36 b C	833.54 ± 4.42 a B
		6 d	748.03 ± 4.60 b A	866.31 ± 7.07 a A
	INIA P708	1 d	717.60 ± 4.94 b B	851.88 ± 3.93 a A
		6 d	757.96 ± 2.10 b A	860.16 ± 5.72 a A
	INIA P334	1 d	714.77 ± 15.98 b A	852.38 ± 14.76 a A
		6 d	741.57 ± 9.40 b A	870.41 ± 4.07 a A

INIA TAB84	1 d	700.11 ± 13.34 $^{b\,B}$	820.26 ± 19.33 $^{a\,B}$
	6 d	722.06 ± 4.67 $^{b\,AB}$	854.99 ± 2.94 $^{a\,A}$
INIA P508	1 d	694.52 ± 12.05 $^{b\,B}$	834.10 ± 13.72 $^{a\,A}$
	6 d	712.04 ± 9.07 $^{b\,AB}$	854.08 ± 5.31 $^{a\,A}$
INIA P459	1 d	703.32 ± 8.91 $^{b\,B}$	828.50 ± 9.61 $^{a\,B}$
	6 d	711.72 ± 3.72 $^{b\,AB}$	853.23 ± 6.24 $^{a\,A}$
GG	1 d	703.46 ± 8.27 $^{b\,B}$	828.12 ± 1.64 $^{a\,C}$
	6 d	747.04 ± 10.40 $^{b\,A}$	871.51 ± 3.01 $^{a\,A}$

Means ± SD from duplicate determinations in two experiments (n = 4). Means within the same row with different lower-case superscripts differ significantly at $P < 0.01$. Means for the same strain within the same column with different upper-case superscripts differ significantly at $P < 0.01$.

*pH and TPC values of cultures at 0 h were identical to the non inoculated ones (A), and were compared to values at 1 and 6 days.
